# The efficacy of playing a virtual reality game in modulating pain for children with acute burn injuries: A randomized controlled trial [ISRCTN87413556]

**DOI:** 10.1186/1471-2431-5-1

**Published:** 2005-03-03

**Authors:** Debashish A Das, Karen A Grimmer, Anthony L Sparnon, Sarah E McRae, Bruce H Thomas

**Affiliations:** 1 Centre of Allied Health Evidence, University of South Australia, Adelaide, Australia; 2 Women's and Children's Hospital, Adelaide, Australia; 3 Department of Computer and information Sciences, University of South Australia, Adelaide, Australia

## Abstract

**Background:**

The management of burn injuries is reported as painful, distressing and a cause of anxiety in children and their parents. Child's and parents' pain and anxiety, often contributes to extended time required for burns management procedures, in particular the process of changing dressings. The traditional method of pharmacologic analgesia is often insufficient to cover the burnt child's pain, and it can have deleterious side effects [1,2]. Intervention with Virtual Reality (VR) games is based on distraction or interruption in the way current thoughts, including pain, are processed by the brain. Research on adults supports the hypothesis that virtual reality has a positive influence on burns pain modulation.

**Methods:**

This study investigates whether playing a virtual reality game, decreases procedural pain in children aged 5–18 years with acute burn injuries. The paper reports on the findings of a pilot study, a randomised trial, in which seven children acted as their own controls though a series of 11 trials. Outcomes were pain measured using the self-report Faces Scale and findings of interviews with parent/carer and nurses.

**Results:**

The average pain scores (from the Faces Scale) for pharmacological analgesia only was, 4.1 (SD 2.9), while VR coupled with pharmacological analgesia, the average pain score was 1.3 (SD 1.8)

**Conclusion:**

The study provides strong evidence supporting VR based games in providing analgesia with minimal side effects and little impact on the physical hospital environment, as well as its reusability and versatility, suggesting another option in the management of children's acute pain.

## Background

Pain is a noxious stimulus which can be interpreted in many ways by different individuals but as yet the mechanisms by which the body manages it are not completely understood. Pain has been defined by the International Association for the Study of Pain as 'an unpleasant sensory and emotional experience associated with actual or potential tissue damage or described in terms of such damage' [3]. Though it is frequently related to physical causes (such as inflammatory processes and nociceptive transmission of pain messages), the experience is entirely subjective, making objective measurement of pain difficult. [4-6], The way in which pain is perceived depends on many factors, including past experiences, memory, understanding of pain, cultural conditioning, and pain threshold [5,7,8].

Children with burn injuries undergo significant physical and emotional trauma, initially from their injury, and subsequently from the dressing changes and related treatment they undergo throughout the healing phase. The latter is referred to, in this article, as 'procedural pain'. Clinicians involved in the care of children with acute burns use the best available methods to reduce procedural pain. Adequate and appropriate pain management is essential to ensure that symptoms secondary to pain experiences do not become habitual [9]. Moreover, unrelieved pain can produce serious physiological and psychological consequences leading to an increased risk of morbidity and even mortality [10,1]. Therefore pain experiences can significantly impact on immediate and longer-term quality of life and well- being of young people.

The burns service at the Women's and Children's Hospital (Adelaide, South Australia) collaborated with University of South Australia, on this project. Pain management comprises administration of a number of medications, including *analgesics*, *muscle relaxants and hypnotics*. These drugs help in reducing procedural pain experienced by children, however they frequently have unwanted side effects such as drowsiness, nausea, reduced postural control and lethargy [11,8].

Pain management of children in hospitals during dressing changes has been reported as inadequate [1,2] and is often described by children to be the most distressing part of the hospitalisation [12,13]. Procedural pain, experienced by children with burns is often distressing for health professionals and parents. Therefore an investigation into nonpharmacological strategies of pain relief for children is warranted from the perspectives of improving pain management, decreasing incidences of side effects and distress in children, parents and health professionals involved with burns dressing changes.

Virtual Reality (VR) was initially conceived as a tool for pain modulation by Hoffman et al [14-18] who found it to be effective in reducing burns pain in adults [15,16] as well as in other situations to manage pain and phobias [14-18]. In 2003, one of our project teams [19] reported on a single subject, cross over design pilot study at the Women's and Children's Hospital (WCH), Adelaide for a child during rehabilitation following orthopaedic surgery. This pilot study suggested the possible usefulness of VR to modulate pain in children undergoing burns dressing changes.

Why virtual reality? VR can be considered intermediary to reality and computer technology. Owing to its ability to allow the user to immerse and interact with the artificial environment that he/she can visualize, the game-playing experience is very engrossing [14-18,20-22]. VR games are different to other games as they give the user a perception of actually being in a different environment. Visual, auditory and touch sensations can be modified based on the stimuli. The game used in this study was developed by the Department of Computer and Information Sciences, University of South Australia. A number of criterions had to be taken into account when designing the game, keeping in mind the different characteristics of prospective players (gender, age groups, intellectual capabilities), the amount of violence portrayed, the complexity of the game and being aware of the amount of control and functions given to the child. The game designers had to keep the structure of the game as simple as possible with minimal controls, to minimise the physical movements required to play the game.

## Methods

This project was an interdisciplinary and inter-sectorial  collaboration between the Centre of Allied Health Evidence, and the  Department of Computer and Information Sciences, both at the University  of South Australia (UniSA), Australia, and Women's and Children's  Hospital(WCH), Adelaide, South Australia.

### Ethics

Ethical approval for this project was obtained from both the institutions (WCH, Adelaide and UniSA).

### Study sample

All children admitted to one specific ward (Newlands Ward), WCH, aged between 5 and 18 years, having burns to more than three percent of their body surface area, and requiring dressing changes, were eligible for inclusion in the study. Children with burns to their hands, face or head, past history of epilepsy and reduced intellectual capacity were not included, as they would have been unable to appropriately use the VR equipment.

### Informed consent

All eligible subjects were identified by ward staff, and were invited to participate in the project by the project team. Written child and parent consent was obtained at every contact.

### Interventions

The test administrations of routine pharmacological analgesia or routine pharmacological analgesia coupled with virtual reality were randomly assigned to each half of the burns dressing change (removal of existing burns dressings or application of fresh dressings) following a coin toss determining the sequence. The child and parents were given a standard explanation about the VR administration and the VR game. If required, subjects were allowed a short preview to assist them to understand how to play the game.

### VR equipment

**Figure 1:**
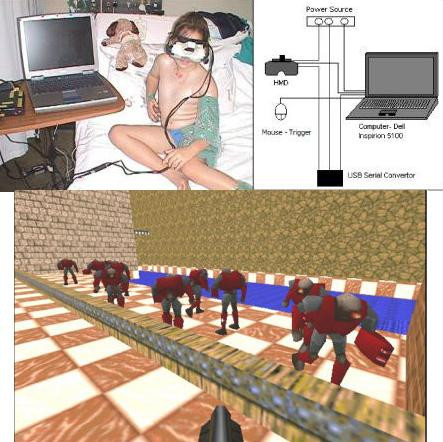
a) Child using the VR equipment, b) Mechanics of the equipment and c) a scene from the game she is playing

• The VR equipment constituted a laptop (Dell Inspiron 5100, Pentium 4 2.4 Ghz CPU with a Radeon Mobility 7500 Video Card) with the game software, developed by the Department of Computer and Information Sciences, UniSA (Based on the game 'Quake' by ID Software), a head-mount display (HMD) (IOGlasses Head Mount Display with a SVGA video resolution of 800 × 600 16 million colours), with a tracking system (Intersense IS300 6 degree of freedom Inertia Cube with a USB-Serial converter, required for Inertia Cube), to allow interaction with the virtual environment by moving the head and neck and decoder and a mouse used as a trigger. The game involved a visual simulation giving the children a feel of being on a track, using a pointer to aim and shoot monsters. Figure 1 illustrates the use of the equipment, and scene from the game.

• The developers considered the applicability of the game through varying age groups, gender, intelligence and intellectual capacities, while designing the game. The game tried to achieve effective distraction via immersion without violence and a simplified game structure requiring minimal control by the player, to allow the smallest possible movement during the dressing change procedure.

### Administration procedure

A within-subject design was used in which the children acted as their own controls.

There was no interference with the dosage or type of analgesia which was administered to the children 30 to 45 minutes prior to dressing removal.

The burns dressing changes would normally occur every Wednesday morning before the hospital ward, grand rounds. The dressing change involved administration of prescribed medication and application of olive oil on the dressing (if adhesive tape was covering the wound), approximately 30 to 45 minutes prior to the actual procedure. The first treatment half constituted the removal of the adhesive tape/bandages and the under-dressing (acticoat/silver oxide dressing) and the second half comprised of the wound being debrided and a fresh dressing applied, after being assessed by the consultant medical officer/s.

### Data collection

**Figure 2:**
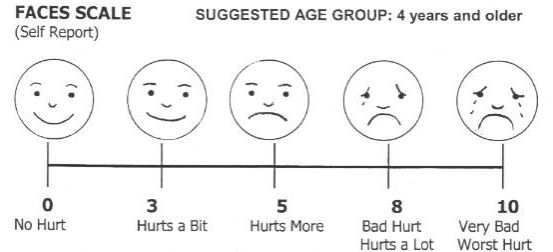
Pain rating scale used by the children [23].

Following the completion of each half of the dressing change (with or without administration of VR), the researcher obtained scores for average pain using the Face Scale (Figure 2), and interviewed the child, mother and the nursing staff regarding their perceptions of the procedure Using standardised questionnaires (Appendix II and Appendix III). One researcher only was involved in data collection, and intra-rater reliability was maintained by using standard protocols for introductions, explanations, VR administrations and data collection procedures.

### Outcome measures

The subjects were asked to score their average pain experience at the end of each phase of the dressing change procedure (VR and pharmacological analgesics, and pharmacological analgesics only). Pain was scored using a modified self-report Faces pain scale [23]. The scale depicts increasing levels of pain and is offered in combination with a visual analogue scale of 0 – 10, associated with each picture representing a level of pain. Parents/carers and nurses were also interviewed by the data collector at these times, using open ended questions to obtain views regarding the child's anxiety and perception of pain, and utility of VR in a clinical setting.

### Data analysis

The data was analysed by a blinded assessor, to reduce any biases and increase the rigour with which a de-identified and coded dataset was probed.

## Results

### Subjects

There were nine eligible, consenting child subjects (6 boys and 3 girls) in the sample. The average age for both boys and girls was 10.0 years (SD 3.7 and 4.1 respectively), age ranging between 5 to 16 years. The average percent of body surface area burnt was 5.3% (SD 3.4%) and there was no significant gender or age difference in body area burnt.

For boys, two had burns from contact with a silencer (muffler) on a four wheeled motor bike, two were burnt from a hot water bag bursting, one was burnt while playing with petrol and fire, and the remaining boy was burnt with hot oil from a BBQ. For the girls, all three were burnt by overturned fluids. All participants were experiencing burns for the first time, and when enrolled into the study, their burns were at either second or third dressing change. Every participants' pain, prior to enrolling in this study, had been managed either with no pain relief, or by pharmacological means.

For every child participant, one parent or guardian with one exception (was not available to observe the dressing change) provided data on the effectiveness of the VR for every post-session interview. One key nurse involved in the burns management was also interviewed following each session.

### Trials

Overall, 13 trials were undertaken from nine children (one subject participating in three trials, two subjects in two trials, and the remainder in one trial each). The results of two subjects were withdrawn for further analysis as the respective participants were too drowsy from the effects of analgesia to participate appropriately in the VR section of the session. Thus the remaining seven child subjects were included for analysis, with a total of 11 useable trials (an average of 1.6 trials per subject). The seven participants in the included trials had an average age of 11.1 years (SD 3.5).

#### Time factor

**Figure 3:**
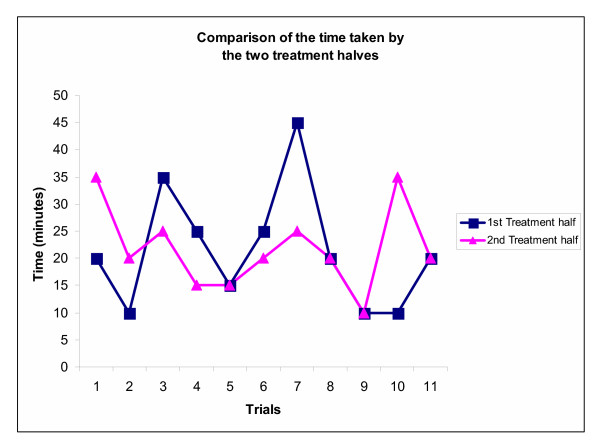
Per trial comparison of time taken to complete each treatment half

There was no significant difference (*p *<*0.05*) in time taken in the two treatment halves (removal and application of fresh dressing). The average difference in administering the two treatment halves was approximately 2 minutes (Figure 3).

### Pain change

**Figure 4:**
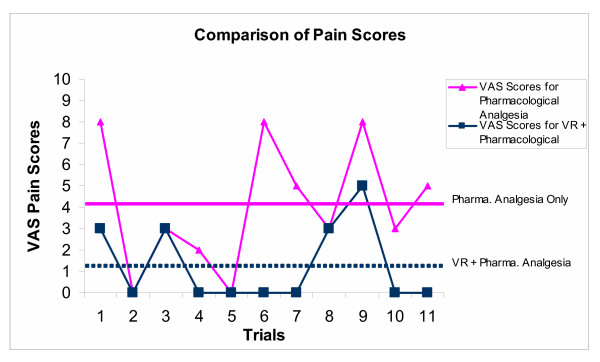
Per trial differences in pain scores compared with average administration scores

With pharmacological analgesia only, the mean pain score (using the Faces Scale), over all included trials was 4.1 (SD 2.9), whilst for VR coupled with pharmacological analgesia, the average pain score was 1.3 (SD 1.8). Because of the small number of child subjects in the study, the data was considered per child, and per trial. Over all included trials, the mean pain score difference between administrations was 3.2 (SD 2.1), which was significant using paired t-tests (p < 0.01). This indicated the importance of the effect of using VR (coupled with analgesia) in reducing pain experiences during burns dressing changes. The per trial pain responses to VR and analgesia, and analgesia alone, compared with the average trial response per administration is shown in Figure 4.

**Figure 5:**
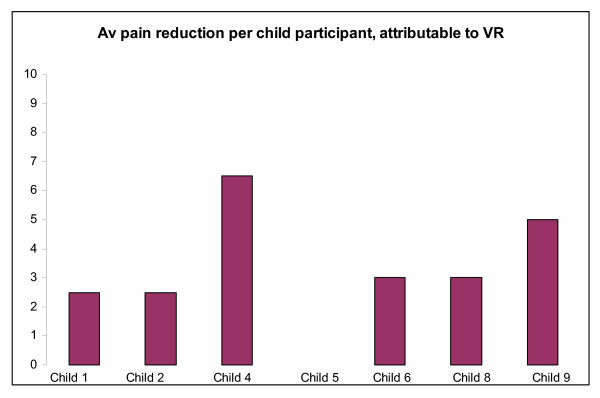
Per child differences in pain response attributable to VR

For each child subject who completed an eligible trial, the average per-child difference in pain scores between administrations of VR & Pharmacological Analgesia, or Pharmacological Analgesia alone, suggested that every child but one obtained an improvement in pain scoring of at least 2 points on the Faces Scale, attributable to VR, as demonstrated in Figure 5.

#### Comments made by nurses, parent/s and child subject

All nurses or parents agreed that VR helped distract the children  and was helpful in reducing pain and there were no negative comments  regarding the application of VR. (note - delete 'interestingly')

### Nurses' responses

The overwhelming response from the nursing staff was that VR administration was helpful to the child. Comments from the interviews are provided below as evidence of this.

"... probably VR helped to take concentration off ... probably helped take away a lot of the anticipation away from the treatment".

*"... from my past experience, I can tell that it *(changing burns dressing) *can be a real problem. It was not a problem today. He did not even flinch while the dressing was being taken off."*

"... communication was good – he understood what I asked him to do. I did not find it invasive or intrusive..."

"... it was great to do the changing (of dressing) without dumping him with medication."

*"... no *(communication was not effected) *... he responded well to the requests and commands."*

*"yes (pain was significantly less)... he had lot more pain with cleaning *(of the wound) *than taking the dressing off... He seemed to cope better with VR than without."*

"... cleaning the dressing in the bathroom made him more anxious, when he saw the wound – felt more pain."

"Yes, he was more anxious when VR was not on."

"... he felt worse when he was looking at it (the wound) compared to when he was not."

"... was more relaxed and concentrated on the game. You could tell that he could feel the pain, but focussed on the game."

The perception of the assisting nurses were the children were more cooperative and distracted from the administration of VR, which helped reduce the difficulty in changing the burns dressing compared to when routine analgesia was used by itself. There appeared to be no problem in physically using VR within the environmental constraints of the burns dressing area, and in no instance did VR impede communication with the child.

### Parents' responses

All parents agreed with the positive effects of VR in pain management for their child. They all commented that the child's anxiety level was perceptibly less when using VR, and the child looked forward to playing the VR game. Comments below from the parent interviews support the positive VR effects:

"... was a lot calmer and enjoyed the VR."

"...absolutely, she did not remember about the last dressing that was taken off. She had to be prompted, to remind her of the game and change of dressing."

"... much happier than usual. He reckoned he felt it but I think he did not. He did not show any of the same signs."

"...she was not as anxious. Was afraid before hand, but she was all right after the game was switched on. It took her mind, off the pain."

"...was smiling while playing the game."

*"... compared to the medication which left him groggy, disoriented, lost track of time and anxious, I think this *(VR) *allows the continuity of time and reduces anxiety."*

*"Yes *(pain was significantly less) *... probably judging it from yesterday – medication made him worse – uncooperative and pig-headed, compared to when he was playing the game."*

"Yesterday he was whinging thinking about the dressing change, this morning, when I told him that you were coming; he had a grin on his face..."

### Comments from child participants on the VR game

However, the current game appeared to have a reasonable level of  complexity and engaged the participating children of different age  groups.

The above comments were randomly selected from the questionnaire deployed at the completion of each trial (to interview the nurses, parents and children participating in the study).

## Discussion

This is the first published randomised clinical trial to our knowledge reporting the use of VR for children with burns. It concurs with the findings of Hoffman et al [15,16] who tested VR on adults with burns, and suggests that VR could provide a significant improvement in the pain management for all children undergoing treatment for this condition. We found that VR coupled with analgesics was significantly more effective in reducing pain responses in children than analgesics only. Although there were 3 occasions where the child, equally scored both treatment halves, the respective carer and nursing staff member, consistently indicated that the child's behaviour was less distressed and calmer during the treatment when VR was applied, suggesting that VR made it less distressing for the child. Thus the feedback given by nursing staff and parents provided additional and important information in interpreting the Faces Scale responses given by the children. Distraction by an interactive game was the putative influence in reducing sensitivity to pain.

### Clinical implications

Given that the application of VR as a method for pain control in the clinical setting is very simple, the results of this study are encouraging with respect to future use. The present prototype VR game being used appears to be cumbersome due to the number of wires attaching to the laptop and the HMD, but this could be simplified considerably so that the equipment required is simply a console with a trigger and a head mount. Applying this equipment would be as simple as providing medication prior to the dressing change procedure. The equipment is reusable and requires minimal technical knowledge for use. Provided a number of different games were available to cater for different age groups, it could be widely applied, and will allow children to relocate themselves to 'another world' during dressing changes, decreasing their attention to painful stimuli. It was noted by nurses during several trials that communication was never a problem; they were able to instruct the child to change, or assist change in position without any difficulty in a compliant and relatively pain-free manner. On the other hand, children without VR were often distressed and crying in pain, decreasing their ability to listen and cooperate.

### Limitations

A number of factors resulted in a small sample size. There were relatively fewer children with burns than anticipated, who fitted the inclusion – exclusion criteria, and who were admitted to the Women's and Children's Hospital, Adelaide, during the data collection period. Several potentially eligible children (2 trials) had such severe side effects from the medication (particularly drowsiness) that they were not able to participate, and two refused to participate. The reduced sample size limits ability to generalise the results, and a study with a larger sample size may provide better understanding of the usefulness of VR as a treatment adjunct for pain relief.

Another potential limitation is that some children were tested more than once – in the first instance immediately after their burn and then subsequently after surgery or during another dressing change later in the healing phase. In these cases, there may have been a learning effect which modified the pain scores, or simply an overall decrease in pain due to healing. It was also noted from the feedback that older children found the game too simple and therefore not as absorbing or distracting as the younger children found it.

Finally, waterproofing of the VR equipment would allow data collectors to examine pain responses through an entire dressing change including when subjects had their wound debridement carried out in the bathroom.

## Conclusion

There appears to be considerable scope for further research into the potential for using VR in the clinical setting. Larger trials could be conducted, using games appropriate for the varying age groups. The next stage would be to test VR alone against pharmacological pain relief, to investigate whether VR is as effective in isolation, and could decrease use of analgesia, thus avoiding the side effects associated with medication. Another avenue of future research would be to investigate the exact mechanisms by which VR assists pain modulation. It is hypothesized that it works by distracting a child's attention from painful stimuli, which in turn reduces the perceived intensity of pain.

## Competing interests

The author(s) declare that they have no competing interests

## Authors' contributions

DAD was involved with coordination and recruitment of trial  subjects, application of the trial, collection of data and writing up  the paper. KAG was involved with statistical analysis of the data and  supervising the scientific conduction of trials. ALS was a key medical  consultant for the trial subjects and coordinated efforts within the  hospital to recruit trial subjects. SEM was the first contact with the  prospective trial subjects. She identified and provided them with  initial information and got verbal consent from them. She also acted as  a coordinator for all the nursing staff members on the ward  participating in the trial. BAT provided IT support for building and  helped in maintaining the inventory (VR equipment) and the game  software.

## Pre-publication history

The pre-publication history for this paper can be accessed here:



## Supplementary Material

Additional File 1 Pain scale scoring Click here for file

Additional File 2 Caregiver interview Click here for file

Additional File 3 Nurse interview Click here for file
