# Supplementary material for: The efficacy of playing a virtual reality game in modulating pain for children with acute burn injuries: A randomized controlled trial [ISRCTN87413556]
Source: BMC Pediatr. 2005 Mar 3;5:1. doi: 10.1186/1471-2431-5-1 (PMC554986; doi:10.1186/1471-2431-5-1)

Patient- pain

**End of 1^st^ half of treatment**

Appendix I

Which picture best shows how the treatment made you feel?


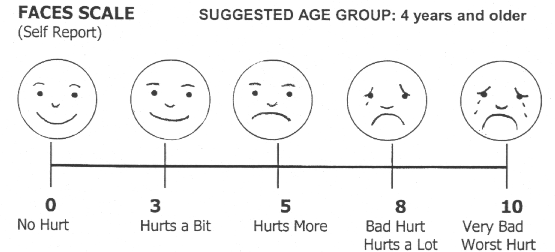


**End of 2nd half of treatment**

Which picture best shows how the treatment made you feel?


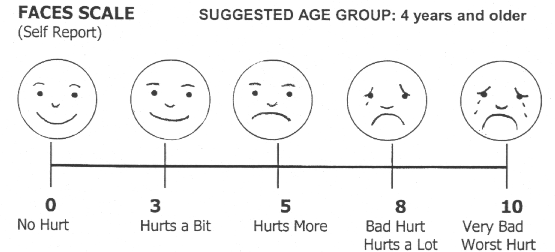

Supplement: Additional File 1 — Pain scale scoring [file 1471-2431-5-1-S1.doc]
