# Supplementary material for: The efficacy of playing a virtual reality game in modulating pain for children with acute burn injuries: A randomized controlled trial [ISRCTN87413556]
Source: BMC Pediatr. 2005 Mar 3;5:1. doi: 10.1186/1471-2431-5-1 (PMC554986; doi:10.1186/1471-2431-5-1)
Supplement: Additional File 2 — Caregiver interview [file 1471-2431-5-1-S2.doc]

**Caregiver interview**

Appendix II

**Caregiver’s assessment of pain modulation effects on the patient**

Interviewer;

Interviewee;

Did you think that (*child’s name)* pain was significantly different when the virtual reality was being used?

Did you think that (*child’s name)* anxiety was significantly different when the virtual reality was being used?

Can you suggest any changes we should make to the way virtual reality is being used? (eg. Changing the equipment)

From what you saw today, do you feel that the use of virtual reality was useful in this setting (burns dressing changes)?
