# Supplementary material for: The efficacy of playing a virtual reality game in modulating pain for children with acute burn injuries: A randomized controlled trial [ISRCTN87413556]
Source: BMC Pediatr. 2005 Mar 3;5:1. doi: 10.1186/1471-2431-5-1 (PMC554986; doi:10.1186/1471-2431-5-1)
Supplement: Additional File 3 — Nurse interview [file 1471-2431-5-1-S3.doc]

**Nurse interview**

Appendix III

**Nurse’s assessment of pain modulation effects on the patient**

Interviewer;

Interviewee;

Did you think that (*child’s name*) level of pain was significantly different when the VR was being used?

Did you think that (*child’s name)* anxiety was significantly different when the virtual reality was being used?

Were there other things besides the use of VR which made the two treatment halves different? What were they? (eg. ‘severity’ of treatment given)

Did the use of VR affect your ability to communicate with your patient? If so, please comment on how greatly you believe this affected your ability to carry out an effective treatment.

Can you suggest any changes we should make to the way VR is being used in this setting? (eg. Changing the equipment)

From what you saw today, do you feel that VR is a useful adjunct to the burns dressing change procedure?
